# Advances in Takayasu arteritis: An Asia Pacific perspective

**DOI:** 10.3389/fmed.2022.952972

**Published:** 2022-08-15

**Authors:** Debashish Danda, Prathyusha Manikuppam, Xinping Tian, Masayoshi Harigai

**Affiliations:** ^1^Department of Clinical Immunology and Rheumatology, Christian Medical College and Hospital, Vellore, India; ^2^Department of Rheumatology and Clinical Immunology, Chinese Academy of Medical Sciences and Peking Union Medical College, National Clinical Research Center for Dermatologic and Immunologic Diseases (NCRC-DID), Peking Union Medical College Hospital (PUMCH), Beijing, China; ^3^Division of Epidemiology and Pharmacoepidemiology of Rheumatic Diseases, Institute of Rheumatology, Tokyo Women's Medical University, Tokyo, Japan

**Keywords:** Takayasu arteritis, HLA B52, ITAS, PET-VAS, PET/MRI, biologicals, granulomatous vasculitis

## Abstract

Takayasu Arteritis (TA) is a rare form of chronic granulomatous large vessel vasculitis that is more common in Asia compared to other parts of the world. There have been several developments in the field of Takayasu arteritis in relation to genetics, classification, clinical features, imaging, disease activity assessment and management and much of these works have been done in the Asia Pacific region. We will be discussing selected few in the current review.

## Introduction

Takayasu Arteritis (TA) is a rare form of chronic granulomatous large vessel vasculitis that is more common in Asia compared to other parts of the world. However the frequency is increasing in other parts of the world, partly due to high immigration rates (1). The Annual incidence rate ranges from 0.4–3.4 per million individuals and the prevalence differs from region to region with Japan having the highest prevalence at 40 per million to 9.0 per million in USA (2). TA commonly presents in second to third decade of life, with a female predominance. Disease may have its onset in childhood and children with TA have significant differences in their clinical presentations from adults (3, 4). Recent studies from Japan and Korea identified differing patterns of arterial involvement in females and males; they found females to have more frequent involvement of thoracic aorta and its branches whereas males were more likely to have abdominal aorta involvement (5, 6).

## Genetics

The first known genetic association with TA was identified by Isohisa et al. (7) who found an increased frequency of HLA-B5 - Bw52 in their 65 patients of Japanese descent. HLA-Bw52 serotype corresponds to HLA-B^*^52:01 genotype in these patients. This was replicated by other independent groups making this association significant in Japanese population (8, 9). The association between HLA-B^*^52:01 genotype and Asian Indians with TA was documented by Rose et al. (10) in 50 patients and Mehra et al. (11) in 80 patients. Two Korean studies (12, 13) and a Turkish study (14) also documented the association in their respective populations. Genetic associations with severity of disease, aortic regurgitation (AR) and glucocorticoid (GC) requirements were also reported for HLA-B52 allele (15).

Chen et al. (16) conducted a meta-analysis with inclusion of 20 studies, 1,864 TA patients and 6,973 controls. HLA-B^*^52 allele was found to be significantly associated with TA with a pooled OR of 3.91. Statistically significant association was also found with TNF-α-308A/G polymorphism.

### Non-HLA and other non Bw52 HLA genes

IL12B region on Chromosome 5 was revealed to have a significant association with TA and the Single nucleotide polymorphism (SNP) rs6871626 in the IL12B region was associated with increased risk and severity of AR (17) and vascular damage (18). Matsumura et al. (19) demonstrated in their study, a strong association between the number of risk alleles in IL12B region and disease severity, pointing to a potential diagnostic use and goal oriented therapy. Yang et al. (20) however did not find any association with polymorphisms of IL 12/23 axis in Chinese population.

An SNP in *IL12B* region (rs755374) was identified as a shared genetic susceptibility loci between Giant cell arteritis (GCA) and TA with the help of Immunochip genotyping data from 1,434 Large vessel vasculitis patients and 3,814 controls (21).

GWAS (Genome-wide association study) in Turkish, North American, Japanese, and Han Chinese patients reported an association with a polymorphism of the genes encoding Fc γ receptor IIA and IIIA (FCGR2A/ FCGR3A) (22). FCGR2A/3A encodes for receptors of Fc portion of immunoglobulin and thereby, is associated with the mechanisms involved in removal of pathogenic antigen antibody complexes (22).

Terao et al. (23) conducted a GWAS study in 633 TA cases and 5,928 controls and reported four previously undocumented loci namely rs2322599, rs103294, rs17133698, and rs1713450, in PTK2B, LILRA3/LILRB2, DUSP22, and KLHL33, respectively. They found a significant epistasis association between HLA-B^*^52 and rs103294 in LILRA3. They also found a novel SNP in the MICB (MHC Class I Polypeptide-Related Sequence B region), which showed a strong linkage disequilibrium with HLA-B52. This SNP contributes to upregulation of MICB on blood vessels, activation of Natural killer cells, and vasculitis.

MLX gene encodes Max like Protein X transcription factor and a single nucleotide polymorphism, rs665268, was associated with severity of disease in the form of AR and the number of arterial lesions. The Q139R mutation in MLX gene leads to suppression of autophagy and enhanced Inflammasome activation leading to cellular oxidative stress (24).

A recently published multi ancestral meta-analysis comprising of TA patients from five different populations (Turkish, Northern European descendant, Han Chinese, South Asian, and Italian), reported independent association with five different variants in HLA region: Three in *HLA-B* susceptibility locus (rs12524487-*HLA-B/MICA*, rs17193507-*HLA-B/MICA*, and rs12526858-*HLA-B*), and 2 other newly described ones namely, rs2844678-*MUC21* and rs28749167-*HLA-G*; in addition, they also identified four non-HLA susceptibility loci in *VPS8, SVEP1, CFL2*, and chr13q21. The study also fortified the association of *IL12B, PTK2B*, and chr21q22 with TA across ancestries (25).

## Pathogenesis

The core of pathophysiology involves inflammation of the aorta, its major branches and coronary and pulmonary vasculature, which leads to arterial wall remodeling and thickening leading to a stenotic or aneurysmal disease (26).

Inflammation begins around the vasa vasorum in early stages of TA, that progresses to cause an inflammatory cell infiltration of adventitia. Eventually, granulomas form adjacent to media, with infiltration of monocytes, lymphocytes, neutrophils, epithelioid cells, and giant cells, eroding the media from outside. Intimal hyperplasia occurs as a consequence of damage to internal elastic lamina by granulomatous inflammation. This, along with adventitial fibrosis leads to stenosis of the arterial lumen causing ischemic symptoms (27). Whereas, a rapidly progressing & aggressive cascade of inflammatory events far overtaking a slower & lagging fibrotic healing process in the vessel wall leads to aneurysm formation (28). The frequency of aneurysms is variable, ranging from 4–45%, among different ethnicities, with highest numbers reported from Japan (29).

### Innate immunity

Vasa vasorum of adventitia is postulated to be the site of primary injury, triggering a sequence of events leading to activation of innate and adaptive immunity (30).

#### Dendritic cells

T cells have been found to co-localize with dendritic cells (DCs) within adventitia in TA (31). Dendritic cells are activated by toll like receptors (TLRs) *via* an unknown trigger. Pryschep et al. (32) demonstrated that there is differential expression pattern of TLRs 1–9 in medium and large vessel walls, which probably explains the pattern of vessel wall involvement in TA.

Immature dendritic cells migrate to secondary lymphoid structures on activation. However, in Large Vessel Vasculitis, the activated DCs produce chemokines CCL18, CCL19, and CCL21 that bind the chemokine receptor CCR7, and get trapped within the vascular wall, stimulating local immunity. They express CD 86 and co-localize with T cells in inflamed vessel walls, indicating their role in linking innate and adaptive immunity (30).

#### Natural killer T cells

Seko et al. (33) demonstrated an increased infiltration of aortic tissue with killer cells, including NK (Natural killer) cells, γδT cells (Gamma delta T cells), and CD8+ T cells. These cells were found to release perforin directly onto the surface of arterial vascular cells. They further analyzed the expression of costimulatory molecules, and found that pro-apoptotic pathways 4-1BB/4-1BBL, MICA/NKG2D and Fas/FasL (Fas ligand) play important roles in vascular wall injury (34).

MICA or Major Histocompatibility Class I Chain-Related A, is induced by cellular stress like bacterial or viral infections. NKG2D, an activating receptor on NK cells and γδT cells binds to its ligand MICA, which initiates cytotoxic responses against the target cells expressing MICA, along with stimulation of antigen specific effector T cells (30).

A recent study demonstrated that TA patients had decreased numbers of NK cells in the peripheral blood compared to healthy controls and the expression of granzyme and perforin was also less compared to healthy controls. This supports the hypothesis that decreased NK cells in the peripheral blood of TA patients is probably due to trafficking to aortic tissues (35).

### Adaptive immunity

#### Role of T lymphocytes

CD4+ Th 1 type cells play a key role in pathogenesis, upon antigen presentation by dendritic cells. These cells promote granuloma formation by releasing interferon-γ (IFN-γ), which in turn activates macrophages and formation of giant cells. Activated macrophages in turn release VEGF (Vascular endothelial growth factor) and PDGF (Platelet derived growth factor) promoting neovascularization and intimal proliferation (36, 37). Studies have demonstrated an increase in activated CD4+ T cells in peripheral blood of TA patients (38) and *in vitro* experiments demonstrated that the peripheral lymphocytes were sensitized against aortal antigen (38, 39).

CD4+ T cells in TA demonstrated ability to spontaneously differentiate into pro inflammatory phenotype. Zhang et al. (40) identified a pathway involving hyperactive mTORC1 (mechanistic target of rapamycin complex 1) leading to spontaneous differentiation into Th1 and Th17 subsets and targeting this pathway in a human artery-NSG (NOD SCID gamma) chimeras led to amelioration of disease.

Role of T cell subsets other than Th1 type in TA is gaining momentum. Weyand et al. (30) demonstrated increase in IL 17 producing T cells before treatment with corticosteroids. Another study from India, recently showed that there is significant expansion of Th17 cells and elevated serum IL-17 and IL-23 levels in 30 TA patients compared to 20 healthy controls (41).

Pan et al. (42) showed elevated IL9 levels in patients with active TA along with increased numbers of Th9 lymphocytes in peripheral blood, suggesting a role in pathogenesis of TA.

Treg cells (T regulatory cells) are key players in maintenance of peripheral tolerance; however under certain circumstances, Treg cells can transform into Th-like cells with loss of tolerance function leading to autoimmunity. Gao et al. (43) have identified increased numbers of Th2 like T regulatory cells in peripheral blood of patients with TA, along with increased levels of IL 4 and IL 13, which correlated with IL 6 levels.

Interferon -γ is a major player in pathogenesis of TA and a recent study by Ren et al. (44) reported that, CD8+ T lymphocytes were a significant source of interferon -γ, with peripheral blood showing higher number of CD3^+^CD8^+^IFN-γ^+^cells and a lower ratio of CD3^+^CD4^+^IFN-γ^+^/ CD3^+^CD8^+^IFN-γ^+^ in TA compared to controls.

There is an imbalance between pro inflammatory (Th1 and Th17) and Treg cells in TA, and upregulated JAK STAT (Janus kinase/signal transducers and activators of transcription) pathway has been identified to have a crucial role. Régnier et al. (45) demonstrated that blockade of this pathway with Jak inhibitors (Ruxolitinib, Baricitinib and Tofacitinib) led to decrease in Th1 and Th17 cells and increase in Treg cells, thereby, restoring homeostasis.

#### Role of B lymphocytes

The role of B cells in pathogenesis of TA is unclear and the evidence is conflicting. A few studies have shown increased frequency of anti-aorta antibodies (46) and anti-endothelial antibodies (47–49) in TA patients vs. controls, however it was not replicated in other studies (50).

Two target proteins, endothelial protein C receptor (EPCR) and scavenger receptor class B type 1 (SR-B1) have been identified for anti-endothelial antibodies in TA. These proteins have anti-inflammatory properties, including impairment of Th 17 function, hence blocking antibodies against them promotes a pro inflammatory state (51).

Tertiary lymphoid structures were demonstrated by Clements et al. (52) in aortic adventitial tissue, however their role in pathogenesis of TA needs to be further studied.

In comparison to Giant cell arteritis patients and healthy controls, TA patients had an upregulated T follicular helper cell signature in another study, which help B cells proliferate and differentiate to produce antibodies. This study also found higher frequency of Tertiary lymphoid structures in inflamed aorta of TA vs. GCA supporting a role for B cell in vascular inflammation (53).

#### Role of pro-inflammatory mediators

Patients with TA have been found to have increased serum levels of IL-6, which correlates with disease activity (54). Seko et al. (55) on amplification of cDNAs (complementary DNA) from the aortic tissue for cytokine transcripts, found increased levels of IL-6 expression in aortic tissue, suggesting an intravascular synthesis. IL 6 has multiple roles, as it activates B cells, enhances T cell cytotoxicity, promotes NK cell activity, activates MMPs (matrix metalloproteinases), fibroblast proliferation and acute-phase protein synthesis (30).

Goel et al. (56) analyzed the serum cytokine profile in Asian Indian patients with active vs. stable Takayasu arteritis and revealed higher serum IFN-γ in active disease than stable disease, and cytokines like Interleukin (IL)-6, IL-23, IL-17, IL-10 and transforming growth factor- β levels did not differ between both groups.

In addition, other cytokines found elevated in TA were IL-8, IL-9, IL-17, IL-18 and TNF (Tumor necrosis factor) (57).

### Triggers of inflammation–environmental factors

The triggering event that leads on to a persistent inflammation of vascular walls is largely unknown. Molecular mimicry to certain peptides of micro-organisms has been suggested as one of the mechanisms. The most notable evidence available is for mycobacterial peptides—mycobacterial heat shock proteins (HSP). Aggarwal et al. (58) reported a heightened response to mycobacterial 65kDa HSP protein in TA patient's vs. healthy controls implying a role for this antigen in pathogenesis of TA. Chauhan et al. (59) demonstrated that there was increased cellular and humoral response to mycobacterial mhsp65 and its human analog Hhsp60, with higher CD4+ T cell proliferation and increased IGG isotype to both antigens, suggesting an infection induced autoimmunity. The link between TA and Tuberculosis (TB) has been hypothesized, due to higher prevalence of TB in Asia, Africa, and South America; the same geographic locations are also home to TA more than rest of the world. In addition, TA is also a granulomatous disease like TB and there have been incidences where TA and TB have occurred together in the same host (30). However, Arnaud et al. (60) did not find any evidence of mycobacterium infiltration in tissue biopsies of TA patients, although this does not rule out a cross reactivity with mycobacterial antigens.

## Disease classification

TA has been traditionally classified into subgroups, based on angiography findings. Most frequently used classification is the Numano classification (also known as Hata's classification) which divided TA patients into six subgroups based on involvement of aorta, as a whole and its main branches (61).

Recently Goel et al. (62) using computer-based cluster analysis strategy, classified TA patients into 3 different subsets based on angiographic patterns, in an Indian cohort of 581 patients. These findings were replicated in 3 independent cohorts from North America. Cluster 1 predominantly involved abdominal aorta and its branches including mesenteric and renal arteries. Cluster 2 had high prevalence of disease affecting aortic arch and its branches and Cluster 3 had asymmetrical arterial involvement with less territorial involvement compared to other clusters. They also observed that out of 92 patients followed up with angiogram, 91 of them stayed in the initial cluster over a median time interval of 3.3 years. Comparison with Numano classification revealed that all three clusters could be represented by Type 5 and 5 % of Indian and 6.7% of North American cohorts were not classifiable according to the Numano classification.

## Clinical features

The clinical spectrum of TA is quite heterogenous, ranging from asymptomatic and incidentally detected hypertension to acute-onset stroke or cardiac failure (3). TA can progress from a prepulseless phase with non-specific systemic features to vascular inflammatory phase, which rarely ends in burnt out fibrotic stenotic phase. However, patients may not conform to this triphasic pattern of disease (3, 63), as ongoing inflammation & damage are the rules rather than exceptions in vast majority of cases with TA in absence of immunosuppressive therapy.

Clinical presentation depends on the pattern of arterial involvement, which is different in different regions of the world. Moriwaki et al. (64) retrospectively compared the clinical manifestations of TA in India and Japan. This study with 102 Indian and 80 Japanese patients documented significant differences in clinical presentation. Indians were most likely to have Type IV disease, with hypertension being the most common clinical manifestation. Japanese TA patients predominantly had type I and IIa disease, with a more severe and prolonged course than Indians.

Goel et al.'s (62) recent work on cluster analysis based angiographic classification revealed that Indians more commonly had renal involvement and predominantly belonged in Cluster 1, whereas most of the North Americans had involvement of left carotid and subclavian arteries and fell within the cluster 3.

Clinical features from various cohorts in the world have been presented in Table 1.

**Table 1:** Clinical features of patients with TA in large cohorts across the world.

**Clinical features**	**India Danda et al. (65)**	**Japan Watanabe et al. (5)**	**China Yang et al. (66)**	**Mexico Soto et al. (67)**	**France Comarmond et al. (68)**	**Turkey Bicakcigil et al. (69)**	**US Schmidt et al. (70)**
**Number of centers**	Single center	Multicenter	Single center	Single center	Single center	Multi center	Single center
**Number**	602	1,372	566	110	318	248	126
**women n (%)**	466 (77.4%)	1,150 (83.8%)	448 (79.2%)	94 (85%)	276 (86.8%)	221 (89.1%)	115 (91%)
**Age of onset**	26 (21–33)^§^	35 (22–56.8) ^§^	36.13 ± 13.4^¥^	26 ± 9^¥^	36 [25–47] ^§^	40.1 (19–76) ^§^	29.2 (20.5–34.5) ^§^
**Fever**	119 (19.2%)	476 (34.7%)	52 (9.2%)	22 (20%)	55 (17%)	68 (27%)	30 (29%)
**Hypertension**	319 (53%)	54 (4%)	62 (11%)	58 (53%)	98 (31%)	106 (43%)	41 (38%)
**Ocular symptoms**	70 (11.7%)	45 (3.3%)	58 (10.2%)	64 (70%)	14 (4%)	57 (36%)	11 (12%)
**Headache**	125 (20.8%)	113 (8.7%)	NA^*^	77 (70%)	NA	119 (48%)	50 (45%)
**Syncope**	66 (11%)	36 (2.6%)	60 (10.6%)	39 (35%)	NA	47 (19%)	51 (49%)
**Stroke**	46 (7.7%)	181 (13.2%)	28 (4.9%)	10 (9%)	39 (12%)	44 (18%)	11 (11%)
**Cardiac** ^*$*^	155 (25.8%)	153 (11.1%)	247 (43%)^*^	35 (32%)	21 (6%)	141 (57%)	39 (39%)
**Lung**	160 (26.7%)	92 (6.1%)	NA	17 (15%)	NA	NA	NA
**Abdominal pain**	NA	NA -	- NA -	NA	NA	NA	15 (16%)
**Renal**	42 (7.7%)	154 (11.2%)	6 (1.1%)	NA	NA	NA	NA
**Carotidynia**	5.6 (33%)	133 (9.7%)	25 (4.4%)	23 (21%)	33 (10%)	NA	15 (15%)
**Limb claudication**	310 (51.5%)	287(20.9%)	161 (28.4)	NA	NA	119 (48%)	64 (52%)
**Pulse abnormalities**	418 (69.6%)	UL 67 (4.9%)	124 (21.9%)	NA	NA	218 (88%)	88 (70%)
**Vascular bruit**	311 (51.7%)	NA	NA	51 (46%)	52 (16%)	190 (77%)	81 (65%)
**Most frequent angiography type**	Type V (51.3%)	Type I (28%)	Type III (37.8%)	Type V (69%)	Type V (49%)	Type V (51%)	Type V (57%)

*NA = “Not available” in the cited reference.

$ *= chest pain, or palpitations, or ischemic heart disease, or aortic regurgitation or heart failure or pericarditis*.

* *Inclusive of aortic regurgitation in the China series*.

§ *= Median with Inter-quartile range*.

¥ *= Mean ± Standard deviation. UL = upper limb*.

In addition to vascular manifestations, extravascular manifestations can also be seen. Most frequently noted features include arthritis/arthralgia with or without sacroiliitis, oral ulcers, inflammatory bowel disease, ocular conditions like scleritis, episcleritis, uveitis, and skin lesions including pyoderma gangrenosum and erythema nodosum (71).

Childhood onset TA, is a subset that can affect any age group ranging from infants to adolescents, with significant differences in clinical presentation compared to adult TA (72). Danda et al. (65) compared clinical characteristics of 108 patients with childhood onset TA (c-TA) vs. 447 patients with adult TA. They observed lesser female predominance, higher frequency of systemic involvement in the form of deranged creatinine, hypertension, cardiomyopathy, abdominal pain, fever and headache in c-TA compared to patients with adult onset TA; the latter were more likely to present with claudication symptoms. When classified according to Hata's angiographic classification, Type IV disease was more frequent in childhood onset TA and Type I was more likely to occur in adult onset TA, although type V was the commonest variety in overall in this series (65).

### Diagnosis

Diagnosis of TA is based on clinical features and supported by imaging and laboratory results. To help differentiate patients with TA, from healthy controls or other similar vasculitic disorders, Ishikawa diagnostic criteria, Sharma's modification of Ishikawa diagnostic criteria and ACR 1990 classification criteria are useful in clinical practice and clinical trials (73). Newly proposed DCVAS-ACR-EULAR classification criteria is awaited.

### Imaging

#### Color doppler ultrasound

With benefits of low cost, easy access, absence of radiation exposure, and ease of repeatability, color Doppler ultrasonography (CDS) has an important role to play especially in a resource-poor setting and in disease assessment in pregnancy. It can detect vessel wall thickening, pulsatility, luminal stenosis, occlusion and calcification (74).

Macaroni's sign is a characteristic feature of TA on CDS, which implies a long segment of moderately echoic circumferential vessel wall thickening which is diffusely homogeneous. Carotid intima-medial thickness (IMT) is useful to assess disease activity with a sensitivity of 82% and specificity of 60%, using common carotid artery wall thickening (75).

CDS has a limited role in assessment of disease in obese patients, and cannot be used to assess origins of greater vessels and abdominal vessels and requires high expertise (74).

#### Contrast enhanced ultrasound

Contrast enhanced ultrasound (CEUS), a technique that is used to assess intraplane neovascularization of carotid artery atherosclerotic plaque, has recently found a place in assessment of disease activity in TA (76). In a retrospective study done on Chinese TA patients, CEUS vascularization score was found to positively correlate with vascular ^18^F-FDG PET/CT (Fluorine-18 - Fluoro-Deoxy-glucose [^18^F-FDG] Positron Emission Tomography [PET]/Computed Tomography [CT]) uptake (77). Ma et al. demonstrated the usefulness of this technique for monitoring response to treatment with reduction in parameters like thickness and vascularization, that were specific to TA (78).

Superb microvascular imaging (SMI), is a novel Doppler technique that can depict low velocity blood flow and microvascular blood flow without the use of a contrast agent (79). This technique has shown arterial wall vascularization in active TA patients, which regressed after treatment suggesting a possible role for it in disease assessment (79, 80).

#### PET-CT

Offering a combination of both anatomical and metabolic imaging, PET-CT has a unique place in diagnosis, especially for patients with atypical presentations, those with normal inflammatory markers (81) and presumably for monitoring of treatment response and predicting relapses (82). In a meta-analysis, from 10 studies, pooled Sensitivity and Specificity of ^18^F-FDG-PET for TA activity was 81 and 74%, respectively (83). It is however influenced by treatment with glucocorticoids, and should not be performed more than 3 days after starting glucocorticoids as it was found to reduce uptake in vessel wall (84). On the contrary, a few studies have suggested that it has a limited value during the follow-up due to persistence of the ^18^F-FDG uptake in a clinically silent disease (85).

PETVAS or PET Vascular Activity Score is a qualitative summary score derived by summation of the semi-quantitative PET visual scores (0 = no ^18^F-FDG uptake; 1 = less than liver; 2 = equal to liver; 3 = greater than liver) of nine specific arterial territories (ascending aorta, aortic arch, descending thoracic aorta, abdominal aorta, innominate artery, right/left carotid arteries, and right/left subclavian arteries). A score of more than or equal to 20 was able to differentiate active from inactive disease with a sensitivity of 68% and a specificity of 71% and was able to predict clinical relapses in a prospectively followed-up cohort. It was also found to have a moderate correlation with acute phase reactants (86, 87).

#### PET/MRI

PET/MRI (Magnetic resonance imaging) combines the quantitative measurement of vessel wall radiotracer uptake of ^18^F-FDG PET along with the anatomic assessment of MRI. This modality has an added advantage of lower radiation exposure compared to PET-CT and hence can be used for follow up scans in younger individuals (88).

Laurent et al. defined three PET/MRI patterns, in their retrospective study of 13 patients with large vessel vasculitis: (a) inflammatory (with abnormal ^18^F-FDG uptake with SUVMAX 4.8 [range: 3–8.6] and abnormal MRI), (b) fibrous (with normal ^18^F-FDG uptake with SUVMAX 1.9 [range: 1.8–2.1] and abnormal MRI), and (c) normal (normal ^18^F-FDG uptake with SUVMAX 2.2 [range: 2–4.5] and normal MRI) (88).

#### MRI

It allows a high-resolution characterization of both vessel wall and lumen, while avoiding radiation exposure. It also has the added advantage of assessing cardiac activity. MRI detects vessel wall thickening and contrast enhancement, that are presumed to reflect inflammation (89). EULAR (currently, European alliance of associations for Rheumatology) task force recommends MRI as a first choice for imaging a patient suspected with Takayasu arteritis (90). T1-weighted, fat-suppressed, contrast-enhanced sequences as well as black blood imaging are preferentially used (91). MRI has a doubtful role in follow up and monitoring of disease activity, as Tso et al. revealed 56% of their study patients in clinical remission had persistent wall oedema and there was no correlation between wall oedema and development of new lesions (92). However, in a study conducted in India in 20 patients with TA, MRI assessment of disease activity significantly correlated with ITAS (Indian Takayasu clinical arteritis score) (93).

#### CT angiography

It is most readily available, preferred diagnostic modality to assess the full extent of arterial involvement, especially when the lumen is unaffected, and the only finding is vessel wall thickening. It has a sensitivity and specificity of 95 and 100%, respectively.

A characteristic feature found in CT angiography (CTA), post contrast is known as double ring enhancement pattern, which is a poorly enhanced ring within indicating a swollen intima and a well enhanced ring outside indicating active inflammation in media and adventitia (94). CTA can depict structural changes including mural thickening, luminal occlusion, aneurysms, thrombosis and end organ damage (95).

CTA is limited by high radiation exposure, restricting its use in follow up and use of contrasts that may have contraindications too. It can delineate anatomical abnormalities, but cannot differentiate between active and inactive disease.

### Biomarkers

CRP (C reactive protein) and ESR (Erythrocyte sedimentation rate) are neither sensitive nor specific for assessment of disease activity, as there can be persistent vessel wall inflammation and angiographic progression of disease in spite of normal acute phase reactants (96). So, there have been a number of studies over time looking for the ideal biomarker for TA.

Goel et al. have looked at the serum cytokine profile and observed that IFN-γ correlated best with disease activity, compared to other cytokines studied including interleukin (IL)-6, IL-23, IL-17, IL-10 and transforming growth factor - β levels (56). They also found a direct correlation between IL-23 and disease duration.

In a Turkish cohort of 51 patients, F. Alibaz-Oner et al. found significantly elevated IL-6, IL-8 and IL-18 levels in patients with TA compared to healthy controls, with higher IL-18 levels in active disease (57).

Nair et al. studied the utility of serum amyloid A (SAA) in assessment of disease activity and observed that SAA levels were higher in TA compared to controls and in active vs. stable disease (97).

Goel et al. explored the usefulness of serial monitoring of serum myeloid related protein 8/14 (MRP8/14) as a marker of disease activity and angiographic progression in (TA). The study revealed that the levels were higher in active disease which decreased in responders to treatment with no change in non-responders. In patients who had angiographic progression on follow up, 66% had elevated MRP8/14 levels compared to 26% in non-progressors. MRP8/14, therefore, could have prognostic implications (98). The same group also looked at soluble HLA E levels as a potential biomarker of disease activity as it is shed from endothelium in response to inflammation. Soluble HLA E levels were higher at baseline and follow up visits of patients with active disease (99).

NMR (Nuclear magnetic resonance) based metabolomics in TA patients revealed a reduced circulatory glutamine to glucose ratio, suggesting increased glutaminolysis and reduced glycolytic activity in active disease which may serve as a surrogate marker for disease activity (100).

The role of B cells in pathogenesis of TA is yet to be explored. A study from India measured levels of APRIL (a proliferation-inducing ligand) and BAFF (B-cell activating factor) in TA patients vs. healthy controls and concluded that serum APRIL levels were elevated in TA patients, but they did not correlate with disease activity (101).

### Assessment of disease

#### Disease extent

***DEI TAK*** (Disease extent index in Takayasu arteritis):

*DEI TAK* is a validated tool developed by Indian Rheumatology Association Core Group for Vasculitis (IRAVAS), based on Birmingham Vasculitis Activity Score (BVAS). It is a weighted score that quantifies the extent of disease at assessment, based on clinical findings only (102). Aydin et al. in their Turkish cohort of patients, found that the agreement between DEI TAK and NIH (National institute of health) criteria was 94% (κ = 0.85). However, the agreement between physician global assessment and DEI TAK was lower when compared to NIH criteria (103). One of the limitations is that, imaging is not taken into account and patients with slow progression of disease did not demonstrate change in DEI TAK (103).

#### Disease activity

To assess disease activity in TA, Misra et al. (104) developed a composite scoring system based on serial assessment of DEI TAK, which allowed selection of items reflecting active disease in recent past (104). ITAS 2010 and ITAS.A with acute phase reactants (ESR or CRP) have been extensively validated (104, 105) with inter-rater variability better than PGA (102). ITAS has been shown to respond to therapy and predict relapses in two different cohorts (106, 107).

#### Disease damage

TADS (Takayasu Damage Score), also derived from DEI TAK is used to assess damage caused by TA. It has 42 items in seven systems, scoring features persistent for at least 3 months. TADS can detect clinically-relevant outcomes, like pulse loss, stent patency and mortality (102). Rajappa et al. in their cohort of 82 TA patients, demonstrated a good correlation between TADS scores and disease duration and higher TADS scores in fatal disease (108). There was a positive correlation between duration of disease and DEI TAK and TADS scores in an Indian cohort of 602 patients with a median disease duration of 32 months for childhood TA and 27 months for adult TA (65).

Ma et al. in their recently published paper, developed a new disease assessment model that combines PET/CT sum of SUV mean, ESR and soluble IL 2 receptor levels. The new model was found to be superior to NIH scoring system in a cohort of 91 Chinese patients. However, this multimodel scoring system needs further validation before its use in clinical practice (109).

## Treatment

### Conventional synthetic DMARDS (Disease-modifying antirheumatic drugs)

#### Methotrexate

A pilot study by Hoffman et al. showed that 80 percent of their cohort of patients (13/16) achieved remission with weekly methotrexate on an average dose of 17mg. However, during a mean follow up of 2.8 years, 44 percent of them relapsed on tapering of glucocorticoids (110).

#### Leflunomide

A prospective open label study from Brazil of 15 difficult-to-treat TA patients, who were started on leflunomide 20 mg per day, had 80 percent of them achieve remission at a follow up period of 9 months. Two patients had angiographic progression on treatment, one of whom was however still in clinical remission (111). An extended follow up study of 12 patients in remission was published in 2016 with a mean follow up period of 43 months, out of which 5 patients remained on leflunomide, rest had to change therapy in view of relapse or adverse drug reactions (112).

Recently, a case series from China by Cui et al. reported a response rate of 83 % at 6 months and 69% at 12 months from a cohort of 56 active TA patients, and at the end of 14 months, 85 percent were still on leflunomide with good tolerability. Also 9 out of 15 patients refractory to cyclophosphamide, responded to leflunomide (113).

#### Cyclophosphamide

A meta-analysis of 10 observational studies, revealed that at least 48 percent of patients on cyclophosphamide achieved partial response (114). A recent study put forth this observation that low-dose cyclophosphamide extended event free survival in a cohort of Chinese patients, and helped improve outcomes in high risk patients (115).

#### Azathioprine

Valsakumar et al. in their cohort of 65 newly diagnosed, treatment-naïve patients, found that a combination of azathioprine and prednisolone brought improvement in clinical symptoms and laboratory markers within 3 months in all patients, and it was observed that the disease was angiographically stable (116).

#### Calcineurin inhibitors

Multiple case reports have shown their efficacy in treating TA, especially in cases associated with pyoderma gangrenosum (117–119).

#### Mycophenolate mofetil

A retrospective observational study from India, with 21 TA patients on MMF noted a significant improvement in disease activity as evidenced by a drop in Median (range) ITAS from 7 (0–19) to 1 (0–7); and these patients had a median follow up duration of 9 months. The authors also reported a significant decrease in glucocorticoid dosage at the last follow up (106). Danda et al. in their cohort of 602 TA patients, had 251 patients with a follow up data of more than 12 months. One hundred and sixty (63%) of these patients had been on MMF. Compared to other DMARDS, numerically higher proportion of patients on MMF had sustained inactive disease, with no serious adverse events (65).

A prospective study from China observed that MMF alone or in combination with glucocorticoids, methotrexate or azathioprine was effective in controlling disease activity and retarding angiographic progression, with an effective rate of 80% (120).

### Biological DMARDS

#### Tocilizumab

A Phase 3 randomized control trial from Japan, The TAKT study, conducted by Nakaoka et al. randomized 36 patients to receive either tocilizumab (TCZ) or placebo with background glucocorticoids. The primary endpoint of time to relapse was longer in the tocilizumab group, even though it barely missed statistical significance (121). In the Long term extension study, with all 36 patients receiving weekly subcutaneous TCZ, most demonstrated clinical improvement, glucocorticoid sparing effect and radiological stabilization at 96 weeks (122).

An observational study by Goel et al. with 10 “difficult-to-treat” TA patients, who had active disease in spite of glucocorticoids and multiple DMARDS with median treatment duration of 27 months, reported that 100 % of the patients receiving monthly intravenous Tocilizumab had achieved clinical response as reflected by ITAS of 0. Six out of ten of these patients maintained response till sixth infusion, with no angiographic progression. They also had an important observation that these patients relapsed on stopping tocilizumab, so the benefit was not sustained (123).

In a systematic review and meta-analysis of observational studies of patients receiving TCZ treatment, it was revealed that 87% achieved at least a partial clinical remission, 88% achieved angiographic stabilization, 62 % had reduced uptake on PET-CT and 94% had reduction in acute phase reactants. Patients on TCZ were able to reduce the median prednisolone dose by 83%. However, the results were heterogeneous (114).

#### Abatacept

In a multicentric double blinded randomized control trial, 36 patients received abatacept at 8 mg/kg, and 26 patients who achieved remission at week 12 were randomized to monthly abatacept or placebo with a background glucocorticoid taper. Primary outcome of relapse- free survival was achieved by 22% in abatacept arm at 12 months compared to 40% by placebo arm. The study revealed that there was no benefit of addition of abatacept to standard regimen (124).

#### TNF inhibitors

Ferfar et al. in their review article, documented 13 studies with 96 TA patients who were treated with TNF inhibitors (infliximab, etanercept and adalimumab). Clinical improvement was reported in 61%, Glucocorticoids were stopped in 39%, and 3 patients showed regression of lesions on MR angiography. Twenty-eight relapses were reported in a follow up period of 24 months (125). A point of concern with TNF inhibitors, however is the high risk of tuberculosis reactivation, especially in endemic countries.

A meta-analysis of 18 observational studies was conducted and the pooled data revealed that, in patients on TNF inhibitors, 81% could achieve partial response in clinical features, and 86 % had angiographic stabilization, with relapses in 32%. Results were however significantly heterogenous across the various studies (114).

#### Rituximab

In a retrospective study of seven TA patients, who were refractory to glucocorticoids and various conventional synthetic and biological DMARDS, Rituximab 2 grams was given as induction therapy followed by maintenance doses every 6 months. Three out of seven patients achieved complete remission, but remaining four patients still had persistent disease and radiographic progression. Results of this study do not support use of rituximab in TA. In contrast to this, prior case reports showed eight out of nine patients had positive results in terms of clinical and imaging criteria (126).

### Jak inhibitors

Upregulated JAK STAT signaling pathways have been reported recently, contributing to the pathogenesis in TA and inhibition of these signaling pathways with Jak inhibitors is a promising new therapeutic avenue (45).

#### Tofacitinib

Multiple Case reports have been published recently describing patients with TA who did not respond to csDMARDs, TNF inhibitors or tocilizumab, had good clinical and imaging responses with Tofacitinib at a dose of 5 mg twice a day (127–130).

An observational study by Li et al. reported 5 consecutive patients with refractory TA, who were started on tofacitinib at 5 mg twice a day; all patients experienced clinical improvement within 4 weeks of starting the JAK inhibitor. Three out of these five patients also had stabilization of radiological disease (131).

Kong et al. in their prospective observational study of 53 patients, compared efficacy of tofacitinib vs. methotrexate (with tapering glucocorticoids in both groups) over a period of 12 months. At 6 months and 12 months, tofacitinib group had a higher complete remission rate and fewer relapses. They did not, however, observe any difference in disease progression on imaging (132).

Two randomized control trials from China, one comparing tofacitinib with adalimumab and the other comparing tofacitinib with methotrexate, are currently ongoing, with results expected in 2025 (133, 134).

#### Upadacitinib

Select–TAK, a phase 3 multicenter randomized control trial, comparing upadacitinib vs. placebo, is currently active, with estimated primary completion date in 2022 (135).

#### Pilot study on ustekinumab

Terao et al. conducted a pilot study and assessed the safety and efficacy of ustekinumab in three TA patients and reported response in clinical symptoms and acute phase reactants. These patients however, did not show any change in vessel wall enhancement on MRI (136).

### Comparative studies

#### Methotrexate vs. leflunomide

When an observational study compared these two drugs over a 12-month duration, there was no difference in the proportion of patients achieving complete remission at 9 months and 12 months. There were however lesser number of relapses in the leflunomide group than in methotrexate group; 7.24% vs. 16.67% (*p* = 0.03) (137).

#### TNF inhibitors vs. tocilizumab

A retrospective observational study, analyzed drug retention rates in 50 TA patients and found that TNF inhibitors had higher drug retention rates than tocilizumab and using concomitant csDMARDs had a positive effect on the same (138).

Another retrospective multicenter study from France investigated outcomes in 49 TA patients and found no difference in efficacy between TNF inhibitors and tocilizumab (139).

Similarly, a multi - center retrospective cohort showed similar remission rates, relapses, glucocorticoid doses and mortality with TNF inhibitors and Tocilizumab (140).

### Endovascular and surgical interventions

The main indications for interventions in TA include critical ischemia with risk of end organ damage, uncontrolled hypertension, coarctation of aorta, aortic aneurysm and aortic regurgitation (141–146).

Interventions should ideally be performed during periods of remission, as the complication rate and mortality is higher when the disease is active (147, 148). Saadoun et al. from their cohort of 79 patients, observed that the likelihood of complications was 7 times higher when procedures were done in the presence of active inflammation (148). A retrospective study by Perera et al. observed that the rates of procedural failure were lower in patients with well-controlled disease (142).

Vascular interventions can be by endovascular approach, or *via* open surgery. A meta-analysis by Jung et al. on 770 patients compared endovascular interventions vs. open surgery. They reported that patients who underwent endovascular interventions had a higher rate of restenosis, especially in coronary artery, supra-aortic branches, and renal artery in both active and inactive disease. The risk of stroke was instead higher in open surgical procedures (149). In a 10-year retrospective study by Diao et al., both types of procedures were found to be safe and had similar primary and secondary patency rates (150).

Joseph et al. published their data on 401 TA patients, who underwent 1,516 percutaneous interventions. Early outcomes were successful in 1,044 interventions. In follow up, on repeated percutaneous interventions in patients who developed restenosis, 83 % success rate was achieved at a mean follow up duration of 33 months from the last procedure (143).

Open surgery that is most commonly performed is the aortic valve surgery with (Bentall procedure) or without aortic root replacement. Other lesions where open surgery is indicated is for repair of thoracic and abdominal aneurysms, although endovascular stents are fast gaining popularity. Surgical bypass procedures are done in occlusive or stenotic lesions (144). Most frequent complications with open surgeries are postoperative bleeding, cerebrovascular accidents, and anastomotic aneurysm (151).

Endovascular interventions, like primary angioplasty can be performed with or without stents. Balloon angioplasty is commonly used in patients with stenotic or occlusive lesions. Stenting is indicated in cases of arterial dissection or persistent stenosis post angioplasty (152). Stents can be drug eluting stents, self-expanding stents or covered stents which are used in aortic aneurysms (153). Thoracic endovascular aortic repair (TEVAR) and endovascular abdominal aorta repair (EVAR) are life-saving interventions in patients with dissections and aneurysms of aorta (154, 155). The efficacy of percutaneous intraluminal angioplasty in renal artery involvement was evaluated by Sharma et al. in 66 TA patients with 96 stenosis. With uncontrolled hypertension being the most common indication, 83 % showed clinical benefit post procedure, however 16 % of them developed restenosis in a median follow up duration of 22 months (156).

Chacko et al. used their in-house developed CO_2_ angiography guided interventions in renal insufficiency patients, where the use of contrast is considered detrimental to the renal functions. The retrospectively attained data showed that the procedure was safe and effective in this subset of patients (157).

In patients with severe renal artery stenosis, who have failed endovascular interventions, renal auto transplantation is a viable treatment option; it is also a great procedure to ameliorate refractory renovascular hypertension in TA in spite of multiple drugs (158).

## Prognosis

Survival rate at 5 years, ranges from 67–100 % in various studies (66, 141, 159). A recent population based study from Korea comprising 2,731 TA patients, the 10 year survival rate was 85% (160). A French multi-center study reported a 96% overall survival rate at 10 years. The survival and mortality rates varied depending on ethnicity and have improved with time (161). Poor prognostic factors affecting survival are progressive disease course, thoracic aorta involvement and retinopathy (68, 161). Mortality rates range from 3–21%, and the most common causes of death are heart failure, stroke, infections and post-procedure complications (162). Goel et al. developed a model using ESR, CRP, Type 4 TA and Low DEI.TAK score, to predict sustained inactive disease with a sensitivity and specificity of 70 and 61.1% respectively. Sustained inactive disease was observed in 34.6% of their patients over the median follow up period of 42 (IQR: 24–81) months (163).

Establishing criteria for remission and introducing Treat to target therapy, could possibly pave the way for better survival. A Treat to Target algorithm developed by Sugihara et al. is a beginning in this direction (164). A practical treatment algorithm proposed by Jha A and Danda D is another recent, flexible and comprehensive therapeutic guide (165).

## Conclusion

Diagnosing TA early remains to be a challenge even today, efforts in this direction for developing new biomarkers are subjects of ongoing research.

With advances in medical and surgical treatments, we hope that the mortality and morbidity of TA can be reduced further by targeted management of disease.

## Author contributions

DD conceptualized the review. PM and DD reviewed the literature and drafted the manuscript. DD, XT, and MH reviewed and edited the manuscript. All authors contributed to the article and approved the submitted version.

## Conflict of interest

The authors declare that the research was conducted in the absence of any commercial or financial relationships that could be construed as a potential conflict of interest.

## Publisher's note

All claims expressed in this article are solely those of the authors and do not necessarily represent those of their affiliated organizations, or those of the publisher, the editors and the reviewers. Any product that may be evaluated in this article, or claim that may be made by its manufacturer, is not guaranteed or endorsed by the publisher.
